# P-1667. Clinical, Laboratory, and Antimicrobial Resistance Patterns in Patients with Diabetic Foot Ulcers during the COVID-19 Pandemic in a Tertiary Care Hospital in Lima, Peru

**DOI:** 10.1093/ofid/ofaf695.1841

**Published:** 2026-01-11

**Authors:** Victor M Llontop-Silva, Camila A Arones-Santayana, Sebastian A Medina-Ramirez, Guiliana Mas Ubillus, Carolina Sarria-Arenaza

**Affiliations:** Universidad Peruana Cayetano Heredia, Lima, Lima, Peru; Universidad Peruana Cayetano Heredia, Lima, Lima, Peru; Universidad Peruana Unión, Lima, Lima, Peru; Universidad Peruana Cayetano Heredia, Lima, Lima, Peru; Universidad Peruana Cayetano Heredia, Lima, Lima, Peru

## Abstract

**Background:**

Diabetic foot ulcer (DFU) is a serious complication of type 2 diabetes mellitus (T2DM), often resulting in infection and amputation. During the COVID-19 lockdown, reduced healthcare access delayed diagnosis and worsened glycemic control, increasing DFU incidence and severity. Antimicrobial resistance adds to the challenge; understanding microbial profiles and resistance patterns is essential for effective treatment. This study describes the clinical and laboratory features of T2DM patients with infected DFUs during the pandemic and evaluates their microbiological and resistance profiles.

**Figure:**
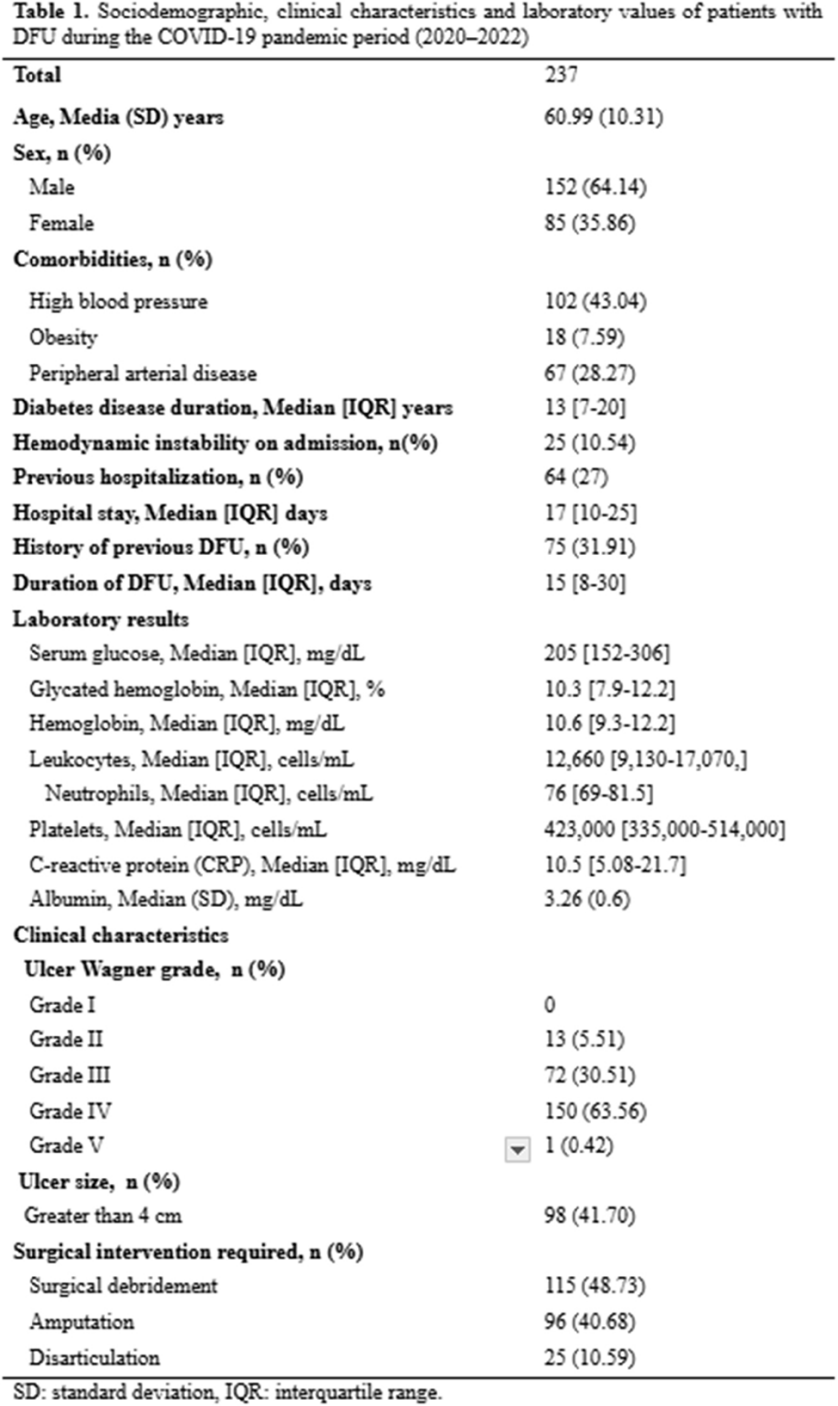

**Figure:**
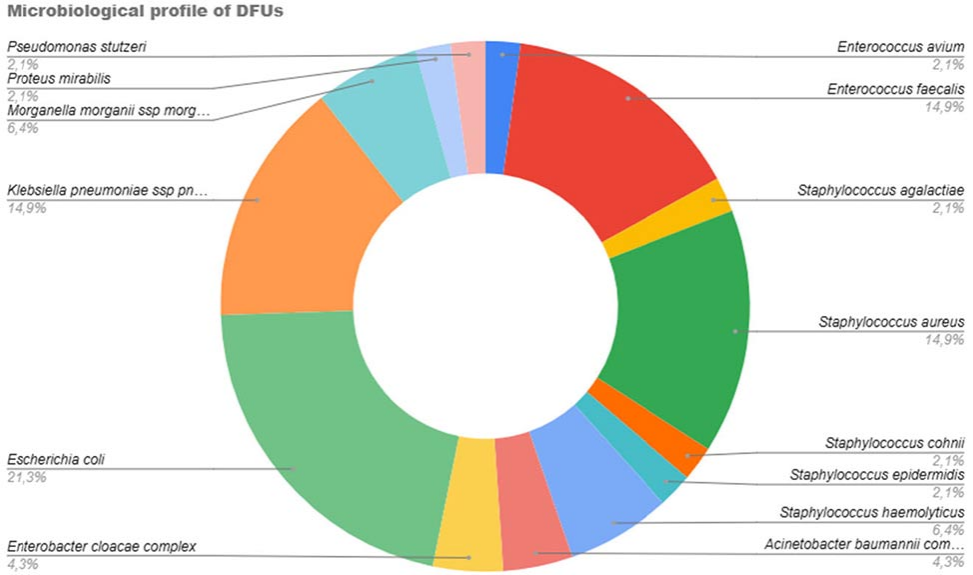

**Methods:**

We conducted a cross-sectional, observational, and retrospective study at Hospital Nacional Arzobispo Loayza, including adult T2DM patients with infected DFU hospitalized in the emergency department between January 2020 and December 2022. Data were extracted from patients' medical records.

**Figure:**
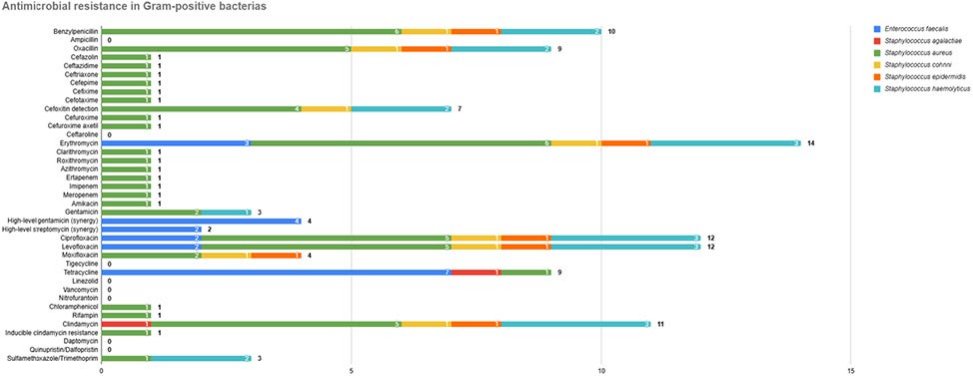

**Figure:**
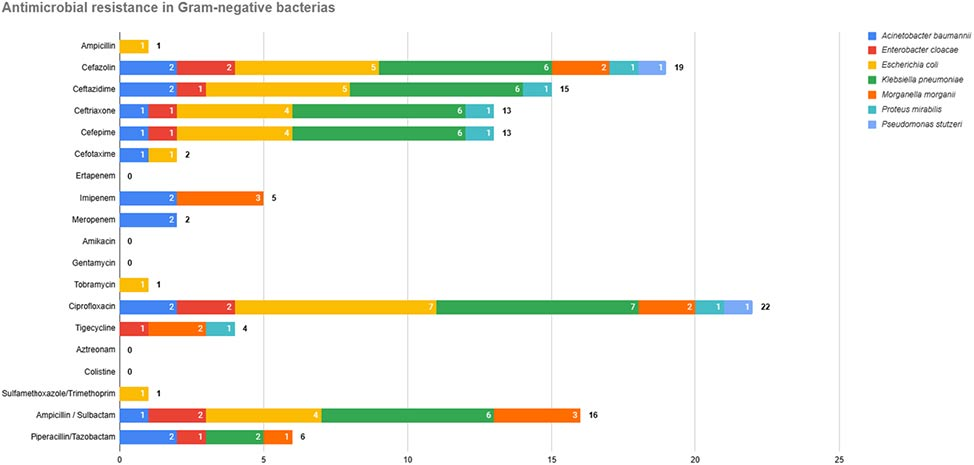

**Results:**

We included 237 patients. The mean age was 61 ± 10.3 years; 64.1% were male. Median glycated hemoglobin was 10.3% [IQR 7.9–12.2]. 68.4% had no history of prior DFU. Wagner grade IV was observed in 150 patients (63.6%). Ulcers > 4 cm were seen in 42.7%. Surgical debridement was performed in 48.7%, and amputation or toe disarticulation in 51.3%. Cultures were obtained in only 14.3% of patients. 55.4% of which were Gram-negative and 35% of these patients had a polymicrobial infection. The most frequent Gram-positive and negative isolates were *S. aureus*, *E. faecalis*, and *E. coli*. Gram-positive organisms showed high resistance to erythromycin (66.6%), tetracycline and clindamycin (52.3%), benzylpenicillin (47.6%), and oxacillin (42.8%). Among Gram-negative isolates, resistance rates were highest for ciprofloxacin (84.6%), cefazolin (73%), ceftazidime (57.7%), and both ceftriaxone and cefepime (50%). MRSA accounted for 71.4% of *S. aureus* isolates. ESBL producers comprised 57.7% of Gram-negatives, predominantly *K. pneumoniae* (85.7%) and *E. coli* (40%), with *E. coli* showing 70% resistance to ciprofloxacin and 40% to gentamicin.

**Conclusion:**

Poor glycemic control, young age, and high amputation rates reflect DFU severity during the pandemic. Despite low culture rates, high antimicrobial resistance highlights the need for routine sampling and surveillance.

**Disclosures:**

All Authors: No reported disclosures

